# Low muscle mass is associated with low insulin sensitivity, impaired pancreatic β cell function, and high glucose excursion in nondiabetic nonobese Japanese women

**DOI:** 10.1016/j.metop.2024.100306

**Published:** 2024-07-30

**Authors:** Satomi Minato-Inokawa, Ayaka Tsuboi-Kaji, Mari Honda, Mika Takeuchi, Kaori Kitaoka, Miki Kurata, Bin Wu, Tsutomu Kazumi, Keisuke Fukuo

**Affiliations:** a Research Institute for Nutrition Sciences, Mukogawa Women's University, Nishinomiya, Hyogo, Japan; b Laboratory of Community Health and Nutrition, Department of Bioscience, Graduate School of Agriculture, Ehime University, Matsuyama, Ehime, Japan; c Department of Nutrition, Osaka City Juso Hospital, Osaka, Japan; d Open Research Center for Studying of Lifestyle-Related Diseases, Mukogawa Women's University, Nishinomiya, Hyogo, Japan; e Department of Health, Sports, and Nutrition, Faculty of Health and Welfare, Kobe Women's University, Kobe, Hyogo, Japan; f Department of Advanced Epidemiology, Noncommunicable Disease (NCD) Epidemiology Research Center, Shiga University of Medical Science, Otsu, Shiga, Japan; g Department of Food Sciences and Nutrition, Mukogawa Women's University, Nishinomiya, Hyogo, Japan; h Department of Endocrinology, First Affiliated Hospital of Kunming Medical University, Kunming, Yunnan, China; i Department of Medicine, Hakuhoukai Kakogawa Hospital, Kakogawa, Hyogo, Japan

**Keywords:** Relative muscle mass, Insulin sensitivity, β-cell function, Glucose excursion, Japanese people

## Abstract

**Aim:**

We tested whether skeletal muscle mass is associated with insulin sensitivity, pancreatic β-cell function, and postglucose glycemia.

**Methods:**

Appendicular skeletal muscle mass (ASM) (relative to body size, %ASM) by DXA, surrogate measures of insulin sensitivity, insulin secretion and the disposition index (insulin sensitivity adjusted insulin secretion: a product of the insulinogenic index and Matsuda insulin sensitivity index) inferred from serum insulin kinetics during a 75 g oral glucose tolerance test (OGTT) were evaluated in 168 young and 65 middle-aged women, whose BMI averaged <23.0 kg/m^2^ and HbA1c ≦ 5.5 %.

**Results:**

In two groups of women, %ASM was associated negatively with homeostasis model assessment insulin resistance (HOMA-IR) and 2-h insulin (both p < 0.01 or less). In middle-aged women not in young women, %ASM was associated inversely with the Matsuda index (p < 0.001). In middle-aged women only, it also showed a positive association with the disposition index (p = 0.02) and inverse associations with 1-h and 2-h glucose (both p < 0.01) and area under the glucose concentration curve during OGTT (p = 0.006). On multivariate linear regression analyses, 2-h insulin emerged as a determinant of %ASM independently of HOMA-IR in young women (standardized β: 0.287, p < 0.001, R^2^ = 0.077). In middle-aged women, the Matsuda index emerged as a determinant of %ASM (standardized β: 0.476, p < 0.001) independently of HOMA-IR, log ODI and AUCg and explained 21.3 % of %ASM variability. Post-glucose glycemia and AUCg were higher and log ODI was lower in middle-aged women with low compared with high %ASM.

**Conclusion:**

Low skeletal muscle mass (relative to body size) was associated with low insulin sensitivity in young and middle-aged Japanese women who were neither obese nor diabetic. Middle-aged women with low muscle mass had low disposition index, an early marker of inadequate pancreatic β-cell compensation, and hence high glucose excursion. Low skeletal muscle mass may be associated with the development of type 2 diabetes at a much lower BMI in Japanese people.

## 1 Introduction

Skeletal muscle is the primary tissue responsible for insulin-dependent glucose uptake in vivo [1] and skeletal muscle insulin resistance is a hallmark of type 2 diabetes [2]. In subjects with preserved pancreatic β-cell function, the relationship between insulin sensitivity and insulin secretion is hyperbolic [3]. Therefore, the product of these two variables, referred to as the disposition index, can evaluate the ability of the β-cell to compensate for insulin resistance.

Some studies in Asians and older normal-weight women [[4], [5], [6], [7], [8], [9]] but not all studies [[10], [11], [12], [13]] demonstrated an inverse association of lean mass with incident type 2 diabetes. Some studies showed an association of muscle mass with insulin sensitivity or resistance [[14], [15], [16], [17], [18], [19]]. However, little is known about the association between muscle mass and pancreatic β cell function.

It is well known that overweight and obesity are associated with type 2 diabetes. Nearly one-third of the world's adult population is overweight or obese [20]. There are ethnic differences in the association between obesity and the risk of type 2 diabetes. Compared with Western populations, Asians are considerably leaner and are more likely to develop diabetes with less weight gain and obesity [21]. Nearly half of people with type 2 diabetes live in Asia, mainly in India and China [22]. Type 2 diabetes is characterized by lower BMI, lower insulin resistance, and lower β-cell function among Indian and Chinese people compared with European people. We have recently reported that adipose tissue insulin resistance was associated with reduced gluteofemoral fat, a subtle partial lipodystrophy-like phenotype associated with reduced adipose tissue expandability, in young lean Japanese women [23]. Lower BMI is associated not only with lower fat mass but also with lower muscle mass. We have recently shown that skeletal muscle mass was inversely associated with an insulin resistance index of adipose tissue in Japanese women [24]. Therefore, the present study tested whether low skeletal muscle mass may be associated with β cell function and glucose excursion in addition to insulin sensitivity/resistance in young and middle-aged non-obese Japanese people.

## 2 Material and methods

### 2.1 Study design, recruitment and participants

We examined cross-sectionally 485 female university students (174 collegiate athletes and 311 nonathletes) and 148 middle-aged mothers whose details have been previously reported [[23], [24], [25], [26], [27]]. Nonathletes were students of the Department of Food Sciences and Nutrition and athletes were students of the Department of Health and Sports Sciences, Mukogawa Women's University. We excluded those with clinically diagnosed acute or chronic diseases, those on hormonal contraception, and those on a diet to lose weight from the study. The present study was done between 2004 and 2007 and these individuals participated as volunteers as previously reported in detail [28].

### 2.2 Ethics clearance and informed consent

This research followed the tenets of the Declaration of Helsinki. All participants gave written informed consent after the experimental procedure had been explained. The study was approved by the Ethics Committees of the Mukogawa Women's University (No. 07–28 on Feb. 19, 2008).

### 2.3 Clinical assessments

Participants underwent blood sampling, measurement of anthropometric indices, and body composition after 12-h overnight fasting. A standard 75 g oral glucose tolerance test (OGTT) was done with multiple post-load glucose and insulin measurements over a 30–120-min period in 168 female students (50 collegiate athletes and 118 nonathletes) and 65 mothers. Blood samples were taken at min 0 (fasting), 30, 60, and 120 for glucose and insulin analysis. Plasma glucose was determined by the hexokinase/glucose-6-phosphate dehydrogenase method (inter-assay coefficient of variation [CV]< 2 %). Serum insulin was measured by an ELISA method with a narrow specificity excluding des-31, des-32, and intact proinsulin (inter-assay CV <6 %).

Insulin resistance/sensitivity was determined by fasting and 2-h insulin [29], homeostasis model assessment (HOMA-IR) using fasting plasma glucose and insulin levels [30] and the Matsuda insulin sensitivity index using glucose and insulin levels during OGTT [31]. Glucose-induced insulin secretion was evaluated by the insulinogenic index (IGI), which was calculated as incremental insulin concentrations (μU/mL) divided by incremental glucose concentrations (mg/dL) during the first 30 min of OGTT [32]. The oral disposition index (ODI) was calculated as the product of the IGI and Matsuda index [33]. The area under the glucose and insulin concentration curve during OGTT (AUCg and AUCi, respectively) was calculated using the trapezoidal method.

Fat mass, lean mass, and total mass of the arms, legs, and trunk in kilograms were measured using whole-body dual-energy X-ray absorptiometry (DXA) (Hologic QDR-2000, software version 7.20D, Waltham, MA) as previously reported [[24], [25], [26]]. Because lean mass in the arms and legs represents skeletal muscle mass, the sum of the two was used as the appendicular skeletal muscle mass (ASM). The ASM index (ASMI) was calculated as ASM in kilograms divided by the squared height in meters. Percentage ASM (%ASM) was calculated as ASM (kg) divided by body weight (kg) × 100. %ASM was used as skeletal muscle mass because % ASM has been suggested to be a better predictor of insulin resistance and diabetes risk than ASM or ASMI [8,34]. General adiposity was assessed by the percentage of body fat and abdominal adiposity by the trunk/leg fat ratio [35].

### 2.4 Statistical analyses

Data were presented as mean ± SD unless otherwise stated. Due to deviation from normal distribution, IGI and ODI were logarithmically transformed for analyses. Pearson's correlation analyses evaluated bivariate correlations of %ASM with anthropometric and metabolic parameters. Stepwise multivariate linear regression analyses were performed to further identify the most significant variables contributing to the variation of %ASM. Independent variables included were variables that showed significant associations with %ASM in Pearson's correlation analysis. Comparisons between the two groups were made with a two-sample *t*-test. Differences among the three groups were analyzed by analysis of variance and then Bonferroni's multiple comparison procedure. A two-tailed p < 0.05 was considered statistically significant. All calculations were performed with SPSS system 23 (SPSS Inc, Chicago, IL).

## 3 Results

### 3.1 Basic demographics and characteristics

ASMI and %ASM were lower in mothers than in female university students including athletic students (Table 1). However, although the differences were small, ASMI was higher in mothers than in non-athletic students (Table 2). ASMI and %ASM were highest in athletic students. All adiposity measures studied were higher in mothers than in students. However, BMI averaged <23 kg/m^2^ even in mothers.

**Table 1:** Anthropometric and biochemical characteristics of young women and their middle-aged mothers.

OGTT	Young women			Mothers		
	n = 485			n = 148		
	n = 168			n = 65		
Age (years)	20.3	±	1.3	49.8	±	3.6 *
BMI (kg/m^2^)	20.9	±	2.2	22.0	±	2.8 *
WC (cm)	72.1	±	5.8	78.7	±	8.1 *
Trunk/leg fat ratio	1.22	±	0.24	1.64	±	0.39 *
% Body fat (%)	26.0	±	5.9	30.1	±	7.3 *
ASMI (kg/m^2^)	6.3	±	0.8	6.0	±	0.5 *
%ASM (%)	30.2	±	3.1	27.5	±	2.9 *
Fasting glucose (mg/dL)	84	±	7	89	±	9 *
2-h glucose (mg/dL)	92	±	21	113	±	28 *
***2-h insulin (μU/mL)***	***36***	***±***	***24***	***37***	***±***	***29***
HbA1c (%)	5.2	±	0.2	5.5	±	0.4 *
***HOMA-IR***	***1.3***	***±***	***0.9***	***1.2***	***±***	***0.7***
IGI	4.6	±	13.0	1.0	±	1.0 *
ODI	43	±	128	9.2	±	10.3 *
***Matsuda index***	***8.9***	***±***	***4.0***	***9.5***	***±***	***4.6***
AUCg (mg/dL/2h)	205	±	41	248	±	56 *
***AUCi (μU/mL/2h)***	***78***	***±***	***36***	***71***	***±***	***38***

Mean ± SD. *: p < 0.05 or less between the two groups. ***Italic bold letters*** indicate no significant differences. AUCg and AUCi, area under the concentration curve of plasma glucose and serum insulin, respectively; BMI, body mass index; % BF, percentage body fat; HbA1c, hemoglobin A1c; ASMI, appendicular skeletal muscle mass (ASM) index; %ASM, percentage ASM: HOMA-IR, homeostasis model assessment-insulin resistance, WC: waist circumference.

**Table 2:** Anthropometric and biochemical characteristics of non-athletic and athletic female university students and mothers.

OGTT	Athletes			Nonathletes			Mothers			
	n = 174			n = 311			n = 148			#
	n = 50			n = 118			n = 65			
BMI (kg/m^2^)	21.6	±	1.9	20.4	±	2.2	22.0	±	2.8	a,c
WC (cm)	74.8	±	5.0	71.2	±	5.7	78.7	±	8.1	a,b,c
Trunk/leg fat ratio	1.17	±	0.21	1.25	±	0.25	1.64	±	0.39	a,b,c
% Body fat (%)	22.8	±	5.1	27.8	±	5.5	30.1	±	7.3	a,b,c
ASMI (kg/m^2^)	7.1	±	0.6	5.9	±	0.5	6.0	±	0.5	a,b,c
%ASM (%)	32.7	±	2.7	28.8	±	2.3	27.5	±	2.9	a,b,c
Fasting glucose (mg/dL)	86	±	6	83	±	7	89	±	14	a,b,c
2-h glucose (mg/dL)	85	±	13	94	±	23	113	±	28	a,b,c
2-h insulin (μU/mL)	23	±	14	42	±	25	37	±	29	a,b
HbA1c (%)	5.2	±	0.2	5.2	±	0.2	5.5	±	0.4	b,c
***HOMA-IR***	***1.2***	***±***	***1.1***	***1.3***	***±***	***0.8***	***1.2***	***±***	***0.7***	
IGI	6.4	±	15.5	3.8	±	11.8	1.0	±	1.0	b,c
ODI	54	±	126	39	±	129	9.2	±	10.3	b,c
***Matsuda index***	***8.5***	***±***	***3.5***	***9.1***	***±***	***4.2***	***9.5***	***±***	***4.6***	
AUCg (mg/dL/2 h)	201	±	38	207	±	42	248	±	56	b,c
***AUCi (μU/mL/2 h)***	***69***	***±***	***25***	***82***	***±***	***39***	***71***	***±***	***38***	

Mean ± SD. Italic bold letters indicate no significant differences. #: p < 0.05 or less by Bonferroni's multiple comparison procedures. a: athletes versus nonathletes, b: athletes versus mothers, c: nonathletes versus mothers. Abbreviations are the same as in Table 1.

Although HbA1c was higher in mothers than in daughters, HbA1c averaged 5.5 % in mothers (Table 1). Fasting and 2-h glucose, and AUCg were higher and IGI and ODI were lower in mothers than in daughters. However, mothers and daughters did not differ in insulin resistance/sensitivity measures (2-h insulin, HOMA-IR, the Matsuda index, and AUCi). When athletic and non-athletic students were analyzed separately (Table 2), 2-h glucose concentrations increased from athletic students through non-athletic students to mothers. The 2-h insulin concentration was lower in athletic students than in non-athletic students and mothers. Incremental glucose concentrations during the first 30 min of OGTT were less than 5 mg/dL in 13 students. Therefore, students had higher IGI and ODI and respective SD than mothers.

### 3.2 Associations of %ASM

Non-athletic and athletic female university students were combined in analyses because association with 2-h insulin did not reach statistical significance when analyzed separately (data not shown). In young and middle-aged women, %ASM was associated negatively with HOMA-IR, fasting, and 2-h insulin (Table 3). In mothers, %ASM was associated inversely with the Matsuda index. It was also associated inversely with fasting, 1-h and 2-h glucose, and AUCg whereas an association between %ASM and fasting glucose was positive in young women. %ASM was associated positively with the ODI in mothers as well.

**Table 3:** Correlation coefficients (r) of percentage appendicular skeletal muscle mass with glucose and insulin metabolism in young women and their middle-aged mothers.

	Young		Middle-aged	
	r	p values	r	p values
Fasting glucose	0**.111**	0**.015**	**−**0**.243**	0**.003**
30min glucose	0.130	0.094	−0.161	0.207
1h glucose	0.022	0.780	**−**0**.346**	0**.005**
2h glucose	−0.118	0.128	**−**0**.367**	0**.003**
Fasting insulin	**−**0**.156**	0**.001**	**−**0**.416**	**<0.001**
30-min insulin	−0.041	0.600	0.038	0.769
1-h insulin	−0.073	0.345	−0.181	0.156
2h insulin	**−**0**.287**	**<0.001**	**−**0**.374**	0**.003**
HOMA-IR	**−**0**.133**	0**.004**	**−**0**.412**	**<0.001**
Matsuda index	−0.065	0.402	0**.476**	**<0.001**
AUCg	0.029	0.708	**−**0**.345**	0**.006**
AUCi	−0.150	0.053	−0.224	0.077
log IGI	−0.038	0.627	0.035	0.788
log ODI	−0.040	0.611	0**.288**	0**.022**

Red figures indicate statistically significant associations. Abbreviations are the same as in Table 1.

In contrast to %ASM, ASMI was associated negatively with the ODI in mothers (Table 4). As expected, % body fat was associated positively with HOMA-IR and inversely with the Matsuda index and the ODI.

**Table 4:** Correlation coefficients (r) of appendicular skeletal muscle mass index (ASMI) and percentage body fat (%body fat) with insulin resistance/sensitivity and β-cell function in middle-aged mothers.

HOMA_IR	ASMI		% Body fat	
	r	p values	r	p values
	0.114	0.175	0.418	<0.001
Matsuda index	0.029	0.820	−0.512	<0.001
log IGI	−0.263	0.037	−0.077	0.551
log ODI	−0.244	0.054	−0.348	0.005

Abbreviations are the same as in Table 1.

### 3.3 Multivariate linear regression analyses for %ASM as a dependent variable

In young women, 2-h insulin emerged as a determinant of %ASM independently of HOMA-IR (Table 5). In middle-aged women, the Matsuda index emerged as a determinant of %ASM and explained 21.3 % of %ASM variability. This association was independent of HOMA-IR, log ODI and AUCg. Including the adipose tissue insulin resistance index [24] as an additional independent variable did not change the results.

**Table 5:** Multivariate linear regression analyses for weight-adjusted appendicular skeletal muscle mass in young and middle-aged Japanese women.

	Standardized β	p values	Cumulative R^2^
Young women			
2-h insulin	−0.287	<0.001	0.077
Middle-aged women			
Matsuda index	0.476	<0.001	0.213

Other independent variables included: young women; homeostasis model assessment-insulin resistance (HOMA-IR), middle-aged women; HOMA-IR, the area under the glucose concentration curve, and log oral disposition index

### 3.4 Features of women with low %ASM

**Fig. 1:**
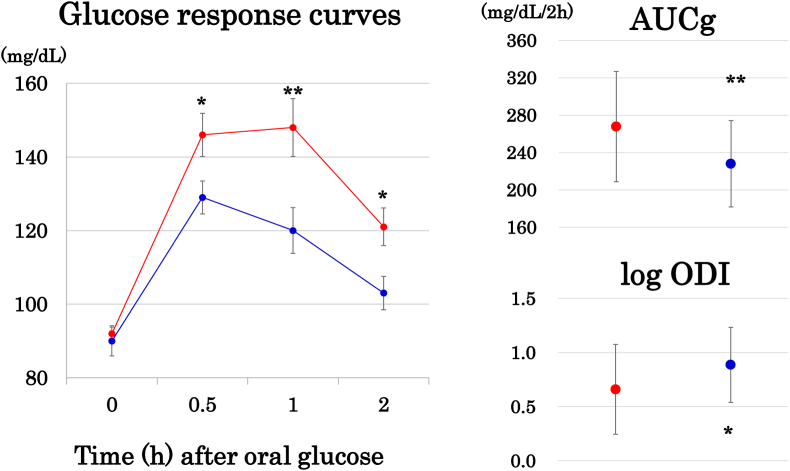
Left column (mean ± SE): Glucose response curves during 75 g oral glucose tolerance testing. Right column (mean ± SD): area under the glucose concentration curve (AUCg) and log oral disposition index (ODI). Middle-aged Japanese women were divided into the high and low groups (blue and red circles, respectively) according to the median value of the weight-adjusted appendicular skeletal muscle mass (ASM) or percentage ASM. *: p < 0.05, **: p < 0.01. (For interpretation of the references to colour in this figure legend, the reader is referred to the Web version of this article.)

**Fig. 2:**
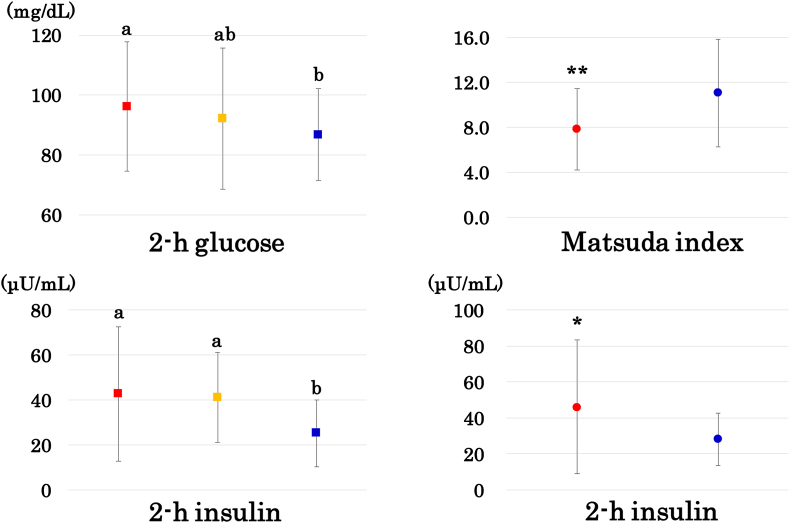
Left column: 2-h glucose and insulin in young women in the low, medium, and high tertile of percentage ASM (red, yellow, and blue squares, respectively). Means not sharing common alphabetical letters are significantly different from each other at p < 0.05 or less by Bonferroni's multiple comparison procedure. Right column: the Matsuda index and 2-h insulin in middle-aged women with low and high percentage ASM (blue and red circles, respectively). *: p < 0.05, **: p < 0.01. (For interpretation of the references to colour in this figure legend, the reader is referred to the Web version of this article.)

Middle-aged women were divided into two groups according to the median %ASM. Post-glucose glycemia and AUCg were higher and log ODI was lower in mothers with low %ASM (Fig. 1). Lower Matsuda insulin sensitivity index and higher HOMA-IR were found as well (Fig. 2). Young women were divided into three groups according to the tertile of %ASM (Fig. 2). Two-hour insulin and unexpectedly, 2-h glucose concentrations were higher in women with the low compared with the high %ASM tertile despite no significant association with 2-h glucose in Pearson's correlation analysis (Table 2).

## 4 Discussion

The present study confirmed associations of low muscle mass (expressed as a percentage of body weight) with low insulin sensitivity (assessed by fasting and 2-h insulin, HOMA-IR, and the Matsuda insulin sensitivity index [[29], [30], [31]]) as previously reported [[14], [15], [16], [17], [18], [19]]. Further, middle-aged Japanese women with low muscle mass had a low ODI, a predictor of future development of diabetes above and beyond fasting and 2-h glucose levels [33], and hence high glucose excursion (AUCg). It is to be noted that these observations were found in women whose BMI averaged <23 kg/m^2^ and HbA1c ≦ 5.5 %.

Little is known about the direct relationship between lean mass and pancreatic β-cell function. To our knowledge, this study is the first to report the negative association of lean mass with pancreatic β-cell function. We found a single study that evaluated insulin secretion by homeostatic model assessment of β-cell function (HOMA-β) and insulin resistance by HOMA-IR in nondiabetic middle-aged Japanese people [36]. Height-adjusted ASM was positively associated with insulin secretion (HOMA-β) and HOMA-IR. However, insulin secretion was not adjusted for insulin sensitivity/resistance.

We have recently suggested that reduced leg fat, a subtle partial lipodystrophy-like phenotype, associated with adipose tissue insulin resistance in Japanese women may be related to the observation that Japanese people tend to develop type 2 diabetes at much lower levels of BMI [23]. The present study suggests that in addition to reduced leg fat, low muscle mass may be associated with glucose dysregulation at lower levels of BMI in Japanese people. These findings may be consistent with our recent report that among body composition measures, leg fat and appendicular skeletal muscle mass were inversely associated with adipose tissue insulin resistance in Japanese women [24].

In subjects with normal glucose tolerance and impaired fasting glucose, insulin-stimulated total glucose disposal, measured with the euglycemic insulin clamp, is strongly and inversely related to the 2-h glucose concentration [37]. Because most glucose disposal during the euglycemic insulin clamp occurs in skeletal muscle, this observation indicates that impaired insulin-mediated glucose disposal in skeletal muscle, i.e., muscle insulin resistance, is strongly related to 2-h glucose concentrations [37]. Therefore, it seems reasonable to assume that an inverse association of %ASM with 2-h glucose in middle-aged mothers in the present study may be mediated by muscle insulin resistance or muscle mass. This may be supported by the stepwise increase in 2-h glucose concentrations from athletic students to non-athletic students to mothers, whose % ASM decreased in a stepwise fashion.

The age-related decline in skeletal muscle mass, referred to as sarcopenia, has been implicated as both a cause and consequence of type 2 diabetes characterized by insulin resistance [38]. Skeletal muscle mass relative to body mass is reduced in the third decade although the absolute quantity of muscle mass is preserved until the fifth decade, with noticeable losses thereafter [39]. %ASM was lower but ASMI was higher in mothers aged 50 years than in non-athletic students. Because our study is cross-sectional, we cannot exclude the possibility that lower muscle mass in mothers may be a consequence of higher HbA1c and glucose excursion for decades. Further longitudinal studies that explore the relationship of higher glucose levels with sarcopenia independent of diabetes are needed.

We found two studies which reported the direct relationship of muscle mass with glucose levels after oral glucose load. In a cross-sectional study of Thai subjects with newly diagnosed type 2 diabetes mellitus and impaired glucose tolerance, total body lean mass measured by DXA was inversely associated with glucose levels after oral glucose load independent of insulin secretion and sensitivity [40]. Higher fasting and post-load glycemia and insulinemia and lower Matsuda index were cross-sectionally associated with lower muscle mass in the Baltimore Longitudinal Study of Aging (mean age 67 years) [9]. In a subsequent study, relatively lower lean body mass with aging was associated with incident diabetes in men, but not so in women [41].

An IGI (μU/mL per mg/dL) of 0.4 and lower is a risk for developing type 2 diabetes in Japanese people with intermediate hyperglycemia and is present in patients with type 2 diabetes in Japan [42]. Although the IGI was lower in middle-aged compared with young women, a mean IGI of 1.1 was much higher than 0.4, suggesting preserved glucose-stimulated insulin secretion in middle-aged women.

The strength of this study includes a homogeneous study population with scarce confounding factors and accurate and reliable measures of body composition by DXA [25]. Several limitations of this study include a relatively small sample size and a single measurement of biochemical variables. We used many surrogates in the present study, which may be less accurate. Statistical power was not calculated. Finally, as we studied Japanese women, results may not be generalized to other sex, races, or ethnicities.

## 5 Conclusions

Low muscle mass (relative to body size) was associated with low insulin sensitivity in young and middle-aged Japanese women who were neither obese nor diabetic. Middle-aged women with low muscle mass had low disposition index, an early marker of inadequate pancreatic β-cell compensation, and hence high glucose excursion. Low skeletal muscle mass may be associated with the development of type 2 diabetes at a lower BMI in Japanese people. Resistance training may be recommended to maintain or increase muscle mass [43].

## Funding

This research did not receive any specific grant from funding agencies in the public, commercial, or not-for-profit sectors.

## Duality of interest

The authors declare that there is no duality of interest associated with this manuscript.

## Data availability

The datasets used and analyzed during the current study available from the corresponding author on reasonable request.

## CRediT authorship contribution statement

**Satomi Minato-Inokawa:** Formal analysis. **Ayaka Tsuboi-Kaji:** Data curation. **Mari Honda:** Data curation. **Mika Takeuchi:** Data curation. **Kaori Kitaoka:** Visualization, Data curation. **Miki Kurata:** Investigation, Data curation. **Bin Wu:** Data curation, Conceptualization. **Tsutomu Kazumi:** Writing – original draft, Supervision. **Keisuke Fukuo:** Writing – review & editing.
